# Utility of the selution SLR™ sirolimus eluting balloon to rescue failing arterio-venous fistulas – 12 month results of the ISABELLA Registry from Singapore

**DOI:** 10.1186/s42155-022-00287-1

**Published:** 2022-02-01

**Authors:** Tjun Y Tang, Charyl JQ Yap, Shereen XY Soon, Ru Yu Tan, Suh Chien Pang, Ankur Patel, Apoorva Gogna, Chieh Suai Tan, Tze Tec Chong

**Affiliations:** 1 Department of Vascular Surgery, Singapore General Hospital, Singapore, Singapore; 2 Duke NUS Graduate Medical School, Singapore, Singapore; 3 Department of Renal Medicine, Singapore General Hospital, Singapore, Singapore; 4 Department of Vascular Interventional Radiology, Sengkang General Hospital, Singapore, Singapore

**Keywords:** Sirolimus coated balloon, Target lesion primary patency, Arterio-venous fistula, Outcome, Safety

*Dear Sir*,

Drug coated balloon (DCB) angioplasty was introduced into the arterio-venous fistula (AVF) arena to offset the neo-intimal hyperplasia (NIH) process and hence reduce the risk of restenosis and prolong access patency (Katsanos et al. 2012). The data regarding the use of paclitaxel-based platforms remain heterogenous to date, despite the presence of level one evidence in the form of randomised controlled trials, and very much depends on the type of paclitaxel balloon utilised and the primary endpoint of interest (Katsanos et al. 2020).

Sirolimus, like paclitaxel, is a potent antiproliferative agent, which has been effective in preventing restenosis in the coronary bed (Ali et al. 2019) and more recently in the peripheral vasculature (Tang et al. 2021). Sirolimus short-term effectiveness and safety in dialysis access circuits has shown early promise in small pilot studies in AVF dysfunction (Tang et al. 2021) and in salvaging thrombosed arterio-venous grafts (Tan et al. 2021). Recently, we had reported 6-month results of the**I**ntervention with **S**elution SLR™ **A**gent **B**alloon for **E**ndovascular **L**atent **L**imus therapy for failing **A**V Fistulas (**ISABELLA)** registry, which was a prospective single-center, multi-investigator, non-consecutive, non-blinded single arm study investigating the safety and feasibility of the *Selution SLR™* sirolimus eluting balloon (SEB) (*M.A. MedAlliance SA*, Nyon, Switzerland) for the treatment of failing AVF in haemodialysis patients (*n*=40) (Tang et al. 2022). The protocol along with novel pre-clinical pharmacokinetic and histological data, to justify its endovascular utility had been recently published (Tang et al. 2021).

All stenotic lesions were prepared with high pressure non-compliant balloon angioplasty prior to SEB angioplasty and lesion effacement and/or recoil < 30% were mandatory in order to be included for subsequent drug elution. All patients received dual antiplatelet therapy for one month and were followed up with Duplex ultrasound at 6 and 12 months. There was one subject dropout so final analysis was based on *n*=39 patients (mean age 65.0 ± 11.9; males = 26 (66.7%)) (Table 1). *N*= 43 target lesions were treated. The most common target lesion was in the juxta-anastomosis (24/43; 54.5%) and 29/43 (65.9%) were recurrent in nature. There was 100% technical and procedural success. There were no adverse events related to the SEB. Target lesion primary patency rates at 6 and 12 months were 28/39 (71.8%) and 16/36 (44.4%) respectively (Table 2). Circuit access patency rates at 6 and 12 months were 22/35 (62.9%) and 10/32 (31.3%) respectively (Fig. 1). Mean time to target lesion reintervention was 6.6 ± 3.7 months with a mean TLR-free duration of 8.6 ± 4.5 months. There were 2 AVF abandonments and 5 (12.8%) deaths at 12 months all attributable to patients’ underlying co-morbidities. 7/10 AVFs re-intervened upon between the 6 and 12-month follow-up timepoints were those with recurrent lesions with an average of 2.9 (± 2.5) reinterventions prior to enrolment into ISABELLA.

**Table 1:** Patient Demographics

	Number of subjects *(n= 39)*	Percentage *(%)*
Mean Age, years (±sd)	65 ± 11.9	
Mean BMI, kg/m^2^ (±sd)	25 ± 4.2	
Gender		
Male	26	66.7
Female	13	33.3
Ethnic Group		
Chinese	28	71.8
Malay	7	17.9
Indian	4	10.3
Smoker	5	12.8
Co-Morbidities (%)		
Hypertension	36	92.3
Diabetes	30	76.9
Hyperlipidemia	27	69.2
Coronary Artery Disease	24	61.5
Cerebrovascular Accident	7	17.9
Medical History		
Beta Blocker	28	71.8
Statin	27	69.2
Antiplatelet	25	64.1
Antidiabetic agents	20	51.3
Warfarin	3	7.7
Access Side		
Left	33	84.6
Right	6	15.4
Access Type		
Radiocephalic	22	56.4
Brachiocephalic	15	38.5
Brachiobasilic	1	2.6
Ulnarbasilic	1	2.6
Median Access Age, months (IQR)	39.5 (18.1-90.6)	

**Table 2:** Patency outcomes

	Number of events (%)	p-value
**6-month patency outcomes**		
Target lesion primary patency *(n=39)*	28 (71.8)	-
De novo *(n=13)*	9 (69.2)	1.00
Recurrent *(n=26)*	19 (73.1)	
JAS *(n=21)*	15 (71.4)	1.00
Non-JAS *(n=18)*	13 (72.2)	
Circuit access primary patency *(n=35)*	22 (62.9)	-
De novo *(n=9)*	7 (77.8)	0.43
Recurrent *(n=26)*	15 (57.7)	
Secondary patency (*n*=36)	35 (97.2)	-
Circuit primary assisted patency *(n=35)*	33 (94.3)	-
**12-month patency outcomes**		
Target lesion primary patency *(n=36)*	16 (44.4)	
De novo *(n=12)*	6 (50.0)	0.73
Recurrent *(n=24)*	10 (41.6)	
JAS *(n=20)*	9 (45.0)	1.00
Non-JAS *(n=16)*	7 (43.8)	
Circuit access primary patency *(n=32)*	10 (31.3)	-
De novo *(n=8)*	3 (37.5)	0.25
Recurrent *(n=24)*	7 (29.2)	
Secondary patency *(n=34)*	32 (94.1)	-
Circuit primary assisted patency *(n=32)*	28 (87.5)	-
Mean TLR-free duration, months (±sd)	8.6 ± 4.5	**-**
Mean time to target lesion reintervention, months (±sd)	7.2 ± 3.6	**-**
De novo	7.1 ± 3.6	0.56
Recurrent	6.4 ± 3.8	
JAS	7.4 ± 4.1	1.00
Non-JAS	5.4 ± 2.6	
Reasons for reintervention		
Dropping access flow	15	
High venous pressure	5	
Cannulation difficulties	1	
Thrombosis	3	
Retrograde flow	1	

Circuit primary assisted patency- freedom from access circuit thrombosis.

Secondary patency – freedom from access circuit abandonment.

JAS; Juxta-anastomotic segment.

**Fig. 1:**
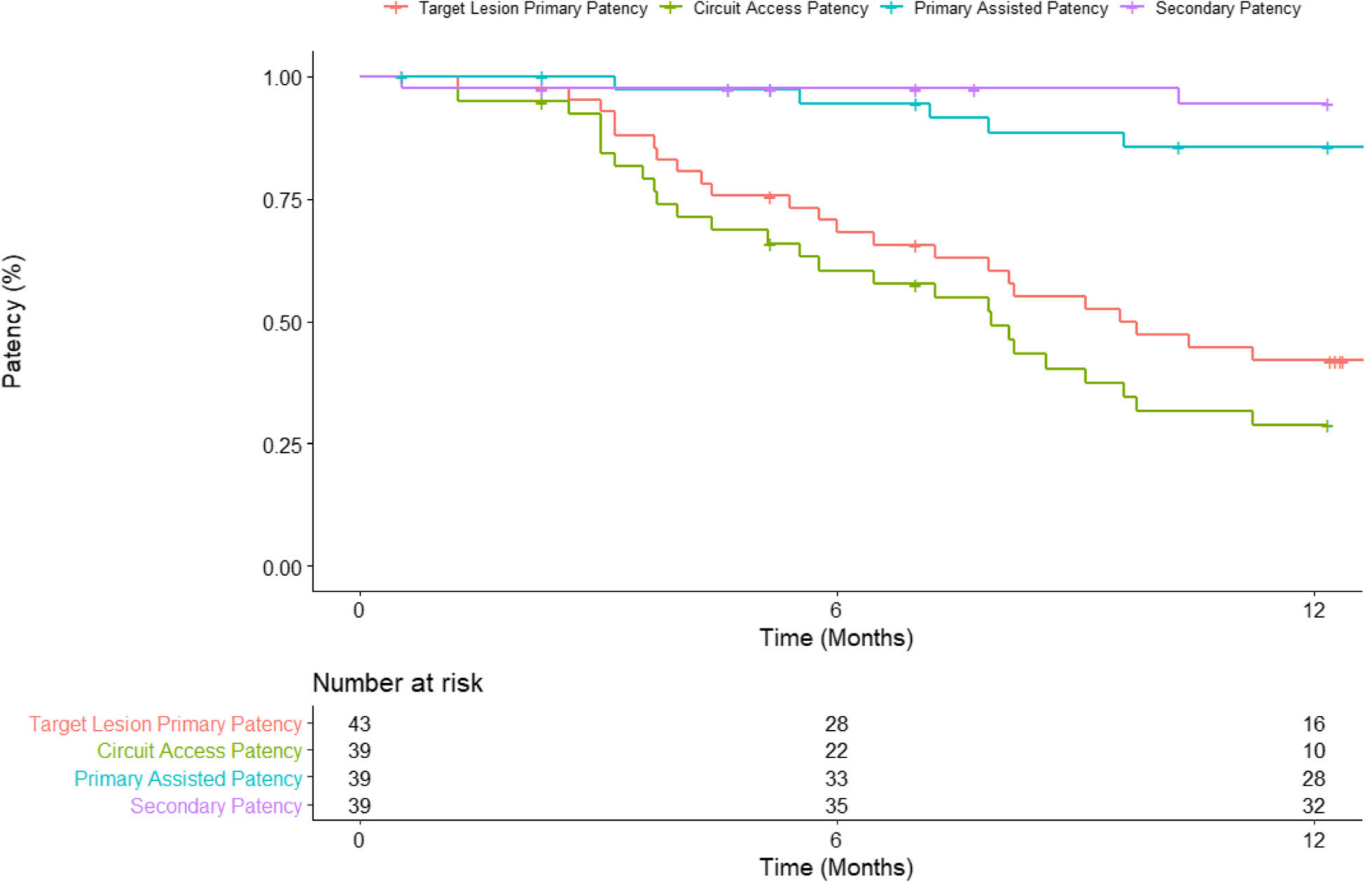
Kaplan-Meier estimates for target lesion primary patency, circuit access patency, primary assisted patency, and secondary patency.

Fistuloplasty using the novel *Selution SLR™*SEB for dysfunctional AVF circuits seems a safe modality in Asian haemodialysis patients at 12 months, with no reported device-related adverse events. Despite initial encouraging 6-months performance results, the drop in TLPP and circuit access patency rates at one year are disappointing but could reflect a need for further drug elution into the adventitial wall to inhibit the NIH process between these two timepoints and the more complex multiple lesions found in Asian AVFs (Irani et al. 2011).

## Data Availability

The datasets generated and/or analysed during the current study are not publicly available due to the Singhealth Centralised Instituitional Review Board requirements but are avaialble from the corresponding author on reasonable request.

## Abbreviations

DCB: Drug coated balloon
AVF: Arterio-venous fistula
NIH: Neo-intimal hyperplasia
ISABELLA: **S**elution SLR™ **A**gent **B**alloon for **E**ndovascular **L**atent **L**imus therapy for failing **A**V Fistulas
SEB: Sirolimus eluting balloon
